# Comparison of hydroxyethylstarch (HES 130/0.4) and 5% human albumin for volume substitution in pediatric neurosurgery: A retrospective, single center study

**DOI:** 10.1186/s13104-021-05836-w

**Published:** 2021-11-27

**Authors:** Martin A. Schick, Jonas Pippir, Manuel F. Struck, Jürgen Brugger, Winfried Neuhaus, Christian Wunder

**Affiliations:** 1 Department of Anesthesiology and Critical Care, Medical Center - University of Freiburg, Freiburg, Germany; 2 Faculty of Medicine, University of Freiburg, Freiburg, Germany; 3 Department for Anesthesiology, Intensive Care Medicine, Emergency Medicine, Pain Therapy and Palliative Care, Klinikum am Steinenberg, Reutlingen, Germany; 4 Department of Anesthesiology and Intensive Care Medicine, University Hospital Leipzig, Leipzig, Germany; 5 Department of Anaesthesiology, Intensive Care, Emergency and Pain Medicine, University Hospital Wuerzburg, Wuerzburg, Germany; 6 Competence Unit Molecular Diagnostics, Center Health and Bioresources, AIT - Austrian Institute of Technology GmbH, Vienna, Austria; 7 Department of Anesthesiology and Intensive Care Medicine, Robert-Bosch-Krankenhaus, Stuttgart, Germany

**Keywords:** Hydroxyethylstarch, HES, Human albumin, Pediatric neurosurgery, Volume substitution, Colloid, Crystalloid

## Abstract

**Objective:**

Colloid solutions are commonly used to maintain perioperative fluid homeostasis. In regard to perioperative infant-centered care, data about the impact of colloids are rare. New data suggest a possible positive effect of hydroxyethyl starch (HES) concerning blood brain barrier. Therefore we conduct a retrospective single center study of children scheduled for neurosurgery, age < five with a blood loss > 10% of body blood volume, receiving either 6% HES 130/0.4 or 5% human albumin (HA).

**Results:**

Out of 913 patients, 86 were included (HES = 30; HA = 56). Compared to HES [16.4 ± 9.2 ml/kg body weight (mean ± SD)] HA group received more colloid volume (25.7 ± 11.3), which had more blood loss [HA 54.8 ± 45.0; HES 30.5 ± 30.0 (%) estimated blood volume] and higher fluid balances. Fibrinogen was decreased and activated partial thromboplastin time was elevated in HA group. Urinary output, creatinine and urea levels did not differ between the two groups. Serum calcium, total protein levels were lower in HES group. HA treated infants tended to have shorter ICU and hospital stays. We conclude that none of the investigated colloid solutions were without leverage to infants. Consequently randomized controlled trials about perioperative goal-directed fluid replacement of children undergoing (neuro)-surgery with major blood loss are needed.

**Supplementary Information:**

The online version contains supplementary material available at 10.1186/s13104-021-05836-w.

## Introduction

Despite numerous studies on perioperative fluid resuscitation, goal-directed fluid therapy (GDFT) remains a daily challenge during surgery. The choice whether to use crystalloids (CL) and/or synthetic or natural colloids (COL) stays controversial [1, 2]. Both hyper- and hypovolemia impairs the patient`s outcome [2]. The comparability of intravenous fluid management between different procedures, e.g. cardiac-, abdominal surgery or ICU, is limited. The number of available data about the impact of HES on children is very low compared to adults’. HES is the most used synthetical colloid and it was established to replace albumin for many circumstances, e.g. for acute hemorrhage. The usage of HES was described as ´safe´ for children [3], although in the recent summary of product characteristics („Fachinformation “) the usage of HES for children is still not recommended. We showed lately, that HES is a nephrotoxic agent and starch even reduced the kidney function in healthy animals, which was only seen in extended kidney diagnostic and histology [4–6]. As another new aspect, we could show that HES might protect blood–brain barrier (BBB) [7]. The detailed mechanism remains unclear. Furthermore, it seems that the risk of HES induced acute kidney injury (AKI) decreased, if HES is used for neurotrauma like stroke or subarachnoidal hemorrhage [8].

The so called Frank Starlings equation (FSE) is the theoretical background for the usage of colloid solutions to maintain hemodynamic stability. FSE implied that the driving force to reabsorb interstitial water back to the blood vessel, is the colloid osmotic pressure, mainly induced by albumin. Both the FSE, as well as the theory for the usage of colloids, are based upon a) a constant pressure in microcirculation and b) sealed microvessels (for macromolecules). However, this does not reflect the situation in critical ill patients (capillary leakage induced by e.g. sepsis) or perioperative setting (rapid vasoconstriction e.g. hemorrhage). Therefore, FSE was revisited by Woodcock et al. and others [9], and FSE theory was extended to reflect clinical settings (e.g. glycocalyx) [9]. Furthermore, Zhang et al. showed, that pediatric patients under the age of six showed no increase in colloid osmotic pressure after HES infusion [10]. Consequently, the question arose: Is there a need for an aged-related, extended Frank Starling Equation?

We conducted a single center, retrospective study to compare HES 130/0.4 with 5% human albumin for volume substitution in children under the age of five with at least 10% blood loss during neurosurgery, to bring more data to the topic synthetical colloids for neurosurgery in children.

## Main text

### Subjects and methods

This study was approved by the institutional review board of the Clinical Ethics Committee of the University of Würzburg, Germany, which waived the requirement for written informed consent because of the retrospective, observational nature of the study. This study included all children (< 5 years of age; n = 913) from 2000 to 2007, who were admitted to the Department of Neurosurgery for neurosurgery. This included craniosynostosis—single suture and syndromic multisuture, crouzon synostosis, tumor (recurrence) with/-out hydrocephalus, tethered spinal cord, cerebral hemangioma, polytrauma with traumatic brain injury. In clinical routine, patients with > 10% of the estimated blood volume (eBV) received infusion of colloids either with HES or HA depending on the respective standard operating procedure for volume- and hemotherapy of the years 2000–2007. Patients with blood loss < 10% eBVwere excluded. The eBV was calculated as 75 ml/kg body weight (BW). Patients were assigned to the HES group ((HES); 6% HES 130/0.4; Fresenius Kabi, Bad Homburg, Germany) or tothe 5% human albumin group (HA), when only HES or HA was infused during surgery and ICU (Fig. S1). The aim was the investigation of sole effects of HES or HA, therefore we excluded patients (n = 39) who received HA and HES together. N = 22 patients were excluded due to incomplete data, n = 2 for other reasons and n = 764 of blood loss < 10% eBV. Finally, 86 children were analyzed. Perioperative blood product transfusion in addition to fluid administration was performed according to the perioperative transfusion protocol of our institution (hemoglobin < 80 g/L and signs of anemia, acute blood loss > 15% of eBV or signs of hypovolaemia, instead of infusion therapy). Primary outcome was defined as total amount of colloids and secondary outcome parameters were length of ICU and hospital stay, ventilation time, coagulation and kidney parameter, blood loss.

#### Statistical analysis

We compared HES with HA. Data derived from continuous variables are presented as medians and interquartile ranges (IQRs) or means ± SD, and data derived from, categorical variables are presented as numbers and percentages. The normal distribution of the residuals was checked to assess the validity of the analysis. Preoperative and postoperative measurements were compared using Student’s paired-t or the Mann–Whitney U test. Kruskal–Wallis test was used for data with an abnormal distribution of variance. The statistical significance of differences between the two groups a p-value of ≤ 0.05 was considered to be statistically significant in all comparisons. Multivariable analysis was not performed due to small sample sizes. Post hoc analysis was not done because it may not provide beneficial additional information. Calculations were performed using SPSS 26.0, IBM.

### Results

**Fig. 1:**
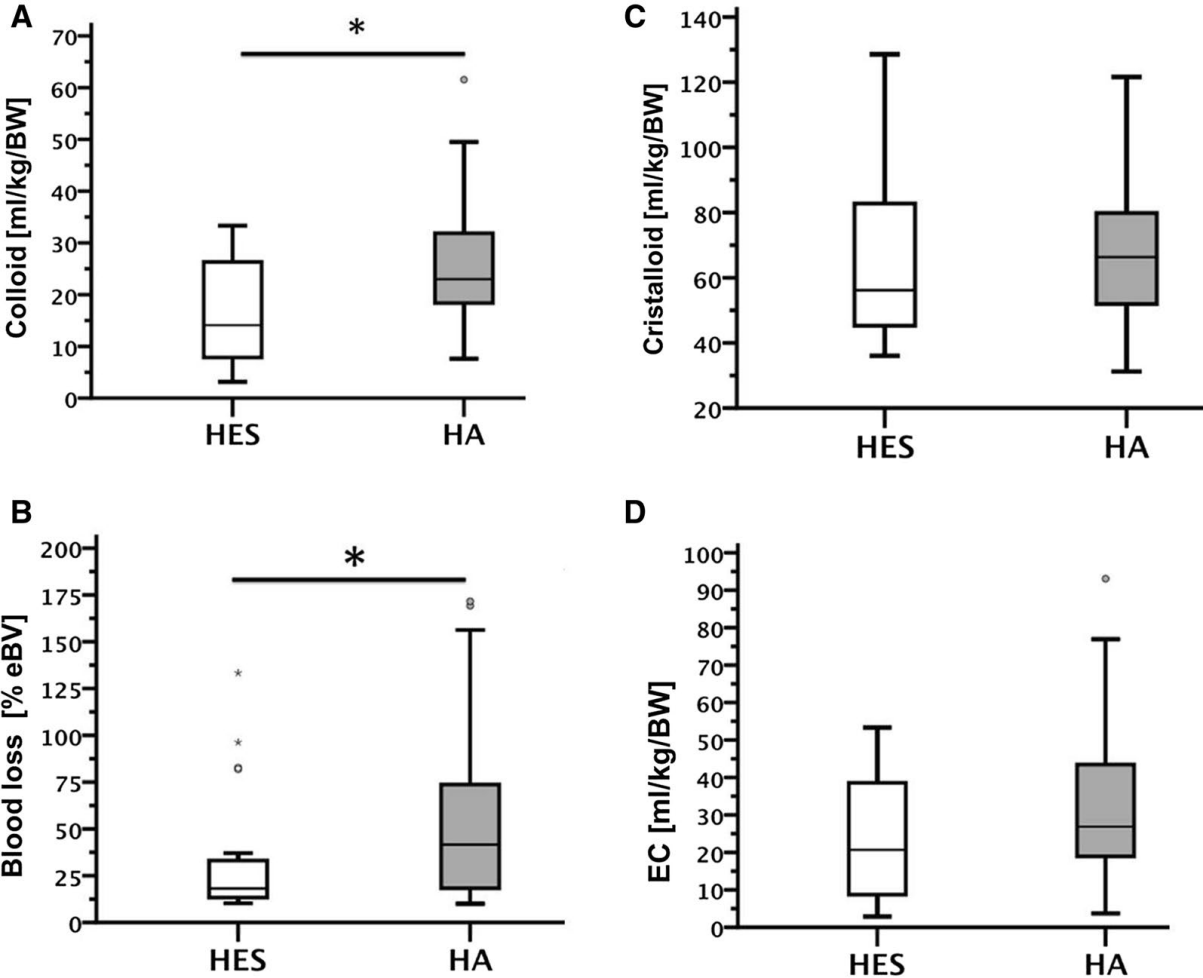
Volume exchange (perioperative data): **A** Cumulative amount of HES or HA (ml/kg/BW). HA was significantly more infused than HES. **C** and **D** No differences were detected of crystalloid infusion and erythrocyte concentrates (EC) (ml/KG/BW). Blood loss in % of estimated blood volume. **B** HA group showed significantly more blood loss compared to HES. HES (6% HES 130/0.4), HA (Human albumin 5%), BW (body weight), eBV (estimated blood volume). *P < 0.05, compared between HES and HA, median ± IQR

From 913 eligible patients, 86 fulfilled inclusion criteria with a documented blood loss > 10% of the eBV (75 ml/kgBW). HA infusion volumes were significantly higher than HES (25.7 ± 11.3 vs. 16.4 ± 9.2 ml/kgBW,mean ± SD, p = 0.001). The gender distribution, weight and surgery duration did not differ between groups (Table S1). The cumulative amount of crystalloids were not different between the groups (HES 66.7 ± 27.5 vs. HA 68.1 ± 21.9 ml/kgBW,mean ± SD, p = 0.967, Fig. 1). HA substituted patients had significantly more absolute blood loss (41.1 ± 33.7 vs. 27.0 ± 31.2 ml/kgBW, p = 0.02) and relative blood loss (54.8 ± 45.0% vs. 30.5 ± 30.0% eBV, p = 0.04) compared to HES. There was a trend toward the length of ICU stay and ventilation time, shorter in HA group and longer when tumor surgery alone was analyzed. ICU stay HES (40.23 ± 25.14) vs. HA (31.6 ± 24.25 [h], p = 0.131), tumor surgery alone HES (54.15 ± 26,34) vs. HA (38,53 ± 26,26 [h], p = 0.066), respectively). ICU ventilation time HES (21.27 ± 16.0) vs. HA (15.61 ± 11.71 [h] p = 0.067), tumor surgery alone HES (29.6 ± 16.8 vs. HA (21.3 ± 14.0 [h]p = 0.127).

**Fig. 2:**
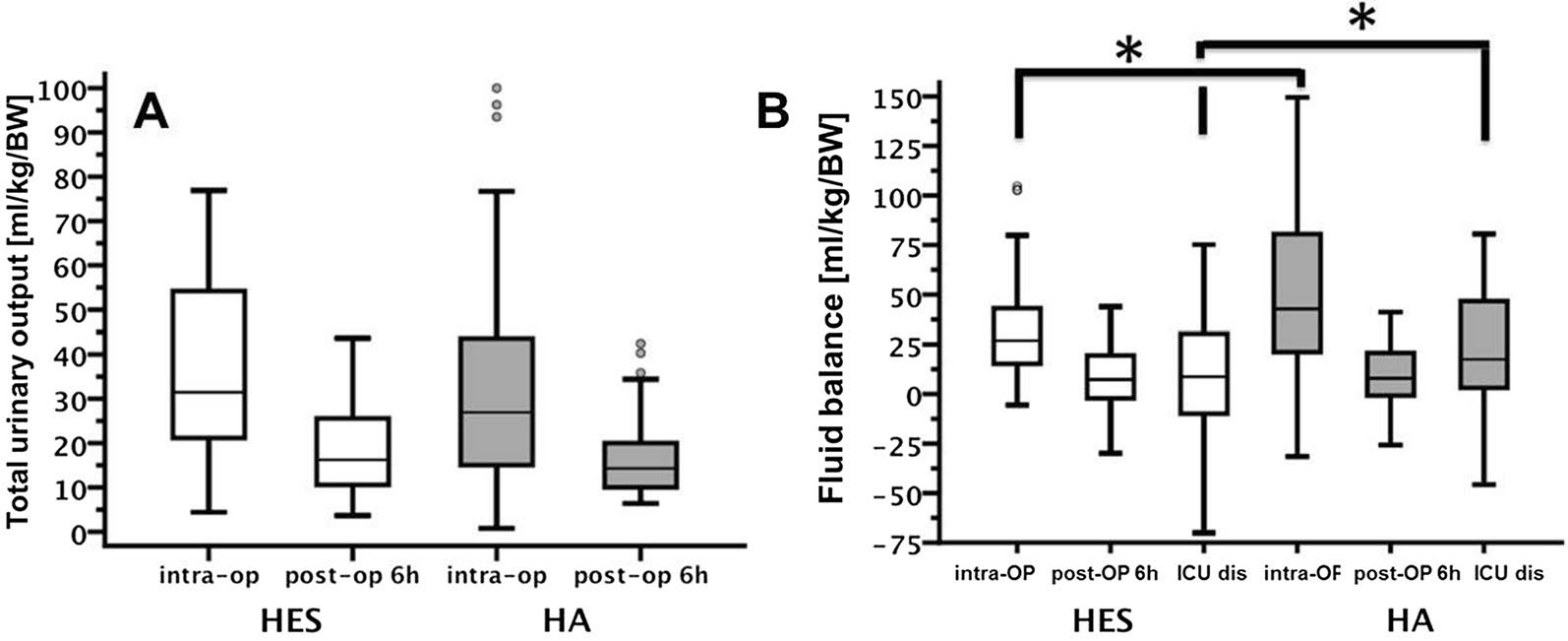
Fluid balance. **A** There were no differences between the groups regarding cumulative urine output (intraoperative and within the first 6 h of ICU). **B** There was a significant positive fluidbalance at the time-point end of surgery (intra-OP) and at discharge from ICU. *p < 0.05 compared between HES and HA

Application of erythrocyte concentrate did not differ between groups (31.9 ± 19.9 vs. 24.0 ± 18.1 ml/kgBW, p = 0.179). HA infusion volumes were significantly higher than HES infusion volumes (25.7 ± 11.3 vs. 16.4 ± 9.2 ml/kgBW,p = 0.001). Urinary output during surgery and within the first 6 h of ICU stay did not differ (p = 0.675 and p = 0.385), whereas fluid balance was significantly increased after surgery and after ICU stay in HA group (p = 0.03 and p = 0.041) (Fig. 2). Also no differences were detected between HA and HES regarding urea and creatinine (p = 0.807 and p = 0.855). Infusion of fresh frozen plasma was not different between groups (24.6 ± 12.0 vs. 35.8 ± 17.2 ml/kgBW p = 0.234), but aPTT was significantly increased in HA (p = 0.006), and Ca^2+^ levels decreased in HES group (p = 0.001). Serum protein level were significantly increased in HA group when compared to HES (p = 0.001) (Table 1).

### Discussion

To our knowledge, the present survey is the study with the most patients comparing HES and human albumin in children with neurosurgery procedures. Our main results show, that children undergoing neurosurgery with major blood loss received significantly more HA compared to HES. HA group had a higher rate of positive fluid balance and decreased coagulation parameters, whereas serum calcium and protein were significantly lower in the HES group.

Clinical trials regarding the use of synthetical colloids for infants are rare. The drawn conclusion, that HES is a safe alternative in the usage in infants, can only be stated by results of adequately powered clinical trials, which are missing. HES could be detected in liver, kidney, skin and immune cells. This may also have beneficial effects, e.g. for the graft kidney [11], despite the knowledge of HES induced AKI. However, the impact of HES on a not fully developed (infant) kidney is not known. To our knowledge, there are no human data available to answer this question. Our presented results showed no differences in standard kidney parameter and urinary output. But in regard to our previous data, this must be examined carefully, since HES induced AKI may only be detected in enlarged kidney diagnostic, such as biopsy and inulin clearance [4]. HA infants showed a positive fluid balance at the end of surgery and ICU stay. We cannot exclude that infused HA had a long lasting extravasal volume effect due to perioperative capillary leakage. Despite the positive fluid balance, the HA group tended to have reduced ventilation time and length of hospital stay, especially in tumor surgery. It was published that HES does not cross the BBB and it is not found in cerebral tissue [8, 12]. However, HES accumulation in destroyed cerebral regions, induced by trauma or surgery, cannot be excluded. Incorporation of HES, in e.g. hypoxic neurons, may have a beneficial effect for survival due to the ability of HES to inhibit formation of reactive oxygen species [8], whereas albumin can be deleterious for astrocyte or neuronal functions by modulating potassium ion and glutamate concentrations, which is driving epileptogenesis [13–15]. HA was associated with higher mortality rates in traumatic brain injury, in contrary HES showed better survival rates (sequential network meta-analyses of 26,351 patients [16]). In this context, the usage of HA for neurosurgery might also be discussed. Due to high cost and limited availability of HA, the need for alternative substances, like HES, remains high. We chose the timeframe for the retrospective study because at that time a maximum dosage of 50 ml/kgBW HES application was allowed. Today’s recommendation by Werner et al. suggests a maximal dosage of 18.8 ml/kgBW 6% HES 130/0.4 in order to prevent the development of HES induced AKI [17]. As expected, in our trial HES was infused up to 33 ml/kgBW, which can result in a high impact on the infants. HA has no recommended maximum dosage, and thus HA was given up to an amount of 62 ml/kgBW in this survey. There were no changes in ventilation time, nor days to discharge after surgery, when the dosage of > 30 ml/kg/BW HA was infused. This raises the questions, whether we need to generally revisit the perioperative usage of HA and, moreover, if we need a maximum threshold adjusted to age. Unexpectedly, serum Ca^2+^ was significantly reduced in HES patients after surgery. This phenomenon is known, but occurs rarely and is hardly described [18, 19]. The serum Ca^2+^ level is in an equilibrium with Ca^2+^ level in the cerebrum. A decreased level of Ca^2+^ in neurons can be protective [8]. Therefore, reduced Ca^2+^ might be an advantage for the cellular survival in the penumbra, but is a disadvantage for the coagulation system since decreased Ca^2+^ levels reduce its efficiency. HES impaired coagulation system in different ways and reduced clot formation in adults as well as in children [20]. Our results showed normal parameters in the HES group, whereas HA application decreased fibrinogen, increased PTT and blood loss. HA can inhibit platelet aggregation or act via heparin-like effects via antithrombin III [21–23]. If HA was used for hemodilution in vitro, it showed hypocoagulation over 25% [24].

### Conclusion

Prospective randomized controlled trials are required to provide evidence-based knowledge on perioperative goal-directed infusion therapy in small children undergoing surgery with major blood loss. Children receiving HA infusion showed decreased coagulation parameters, increased blood loss, increased colloid volumes and positive fluid balance, compared to children receiving HES infusion. Renal function revealed no differences between groups. Our data do not allow recommendations whether to infuse HES or HA during pediatric neurosurgery.

### Limitations

First, despite of 913 patients under the age of five years only 86 could be included with blood loss > 10% eBV and infusion of either HA or HES. This small number of patients, in a single center retrospective observation study, weakens the value of the results. As far as we know, we analysed the most extensive data about volume substitution in pediatric patients receiving neurosurgery procedures. Second, 50% of the performed surgery was tumor surgery, which reduces the overall comparability. Though, the ratio within the two groups (tumor -/ other surgery) was similar [HES 50/50, HA 46/54 (%).] Third, the guidelines for perioperative intravenous fluid therapy for children is permanently changing [25]. However, the results of the survey remain valid, since both substances are still utilized for pediatric patients**.**

**Table 1:** Hematolocical data

		HES		HA	
		Baseline	ICU	Baseline	ICU
Blood count					
hb	[mmol/l]	7.64 ± 1.2*	6.64 ± 0.19	7.33 ± 0.06	6.40 ± 1.2
hct	[%]	35.7 ± 0.7	32.1 ± 0.8	36.7 ± 0.4	32.3 ± 0.6
Leucocyte	[10*3/μl]	9.6 ± 0.5	8.8 ± 0.5	10.6 ± 0.4	8.7 ± 0.4
Platlet	[10*3/μl]	379 ± 22	256 ± 14	401 ± 18	228 ± 11
Coagulation					
PT	[%]	99 ± 1	81 ± 2	97 ± 1	80 ± 2
aPTT	[sec]	34.4 ± 1.2	35.5 ± 1.2*	35.1 ± 0.8	41.2 ± 1.4
Fibrinogen	[mg/dl]	251.1 ± 13.8	189.5 ± 10.6*	262.2 ± 9.7	149.4 ± 6.8
Electrolyte					
Sodium	[mmol/l]	138 ± 1	140 ± 1	138 ± 0	140 ± 0
Potassium	[mmol/l]	4.6 ± 0.1	3.9 ± 0.1	4.6 ± 0.1	3.8 ± 0.1
Calcium	[mmol/l]	2.45 ± 0.04*	1.78 ± 0.89*	2.5 ± 0.2	2.29 ± 0.04
Kidney					
Creatinine	[mg/dl]	0.30 ± 0.02	0.3 ± 0.02	0.31 ± 0.01	0.32 ± 0.01
Urea	[mg/dl]	22.6 ± 2.1	17.9 ± 1.4	23.0 ± 1.9	19.8 ± 2.3
Liver					
ALT	[U/l]	19 ± 1.8	13 ± 1.0*	16 ± 1.0	10 ± 0.6
GGT	[U/l]	16.3 ± 4.2	9.8 ± 1.3	8.5 ± 0.7	9 ± 1.8
Total serum protein	[g/dl]	6.8 ± 0.1	4.5 ± 0.1*	6.6 ± 0.1	5.2 ± 0.1
BG	[mg/dl]	n.a	115 ± 4.8	n.a	110 ± 3.6

Data are mean ± SEM. HES (6% HES 130/0.4), HA (Human albumin 5%) *p < 0.05 compared between HES and HA. *Hb* hemoglobin, *hct* hematocrit, prothrombin time, PT, *ALT* alanine transaminase, *GGT* gamma-glutamyl transferase, *BG* blood glucose

## Supplementary Information

**Additional file 1: Figure S1**. Study population (CONSORT statement). UKW (University hospital of Würzburg), HES (hydroxyethylstarch), HA (human albumin).

**Additional file 2: Table S1**. Population and clinical characteristics (mean±SD).

**Additional file 3**. CONSORT 2010 checklist of information to include when reporting a randomised trial.

## Data Availability

Request to: martin.schick@uniklinik-freiburg.de.

## Abbreviations

AKI: Acute kidney injury
BW: Body weigth
CL: Crystalloid
COL: Colloid
eBV: Estimated blood volume
FSE: Frank Starling equiation
GDFT: Goal directed fluid therapy
HA: Human albumin
HES: Hydroxyethylstarch
ICU: Intensive care unit
