# Supplementary figures and images for: Comparison of hydroxyethylstarch (HES 130/0.4) and 5% human albumin for volume substitution in pediatric neurosurgery: A retrospective, single center study
Source: BMC Res Notes. 2021 Nov 27;14:434. doi: 10.1186/s13104-021-05836-w (PMC8627096; doi:10.1186/s13104-021-05836-w)

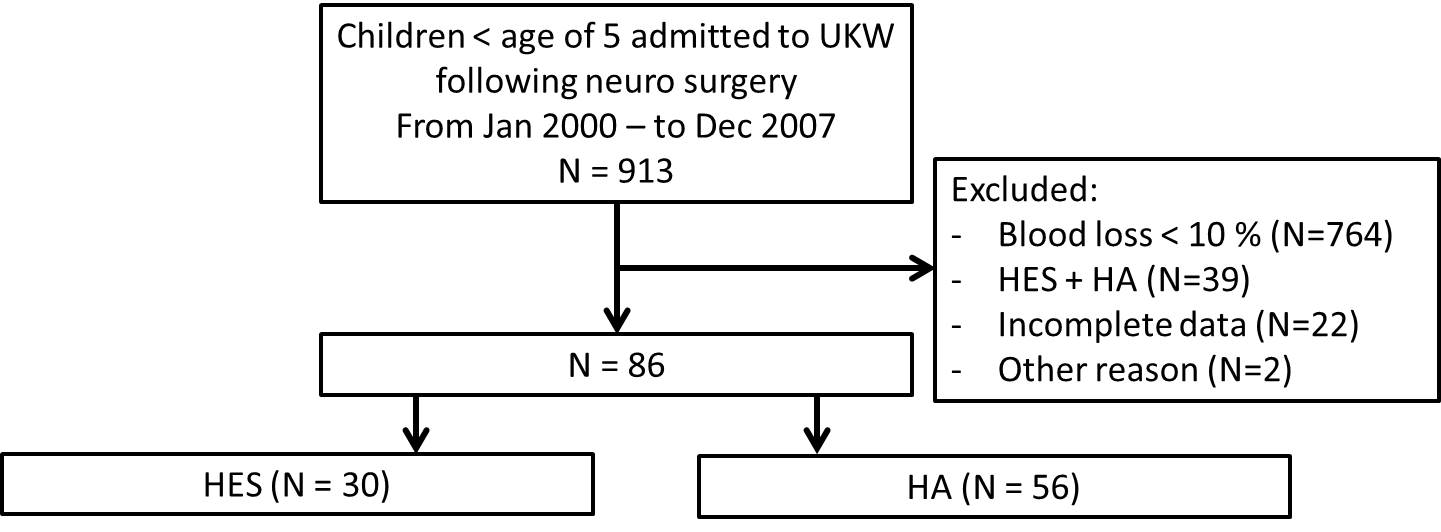

Supplement: Supplementary file 1 — Additional file 1: Figure S1. Study population (CONSORT statement). UKW (University hospital of Würzburg), HES (hydroxyethylstarch), HA (human albumin). [file 13104_2021_5836_MOESM1_ESM.jpg]
