# Supplementary material for: Comparison of hydroxyethylstarch (HES 130/0.4) and 5% human albumin for volume substitution in pediatric neurosurgery: A retrospective, single center study
Source: BMC Res Notes. 2021 Nov 27;14:434. doi: 10.1186/s13104-021-05836-w (PMC8627096; doi:10.1186/s13104-021-05836-w)
Supplement: Supplementary file 2 — Additional file 2: Table S1. Population and clinical characteristics (mean±SD). [file 13104_2021_5836_MOESM2_ESM.docx]

|  | HES | HA | Sign. |
| --- | --- | --- | --- |
|  | N=30 | N=56 |  |
| Gender (F/M; n) | 18/12 | 32/24 |  |
| Weight (kg) | 12.3±4.0 | 11.0±3.9 | P<0.062 |
| Age (mo) | 26.8±17.6 | 20.0±17.9 | P<0.096 |
| ASA 1/2/3/4 (n) | 6/18/5/1 | 21/29/5/1 |  |
| Balanced/ TIVA (n) | 24/6 | 52/2 |  |
|  |  |  |  |
| Surgery duration (h) | 5.29±1.98 | 5.18±2.06 | P<0.985 |
| Ventilation (intraOP+ICU) (h) | 29.59±16.93 | 24.04±12.20 | P<0.066 |
| Length of ICU stay (h) | 40.23±25.14 | 31.60±24.25 | P<0.878 |
| Discharge post OP (d) | 11.0±3.8 | 9.8±4.6 | P<0.209 |
|  |  |  |  |

**Supplemental Table 1.** Population and clinical characteristics (mean±SD).
